# Asperulosidic Acid Ameliorates Renal Interstitial Fibrosis via Removing Indoxyl Sulfate by Up-Regulating Organic Anion Transporters in a Unilateral Ureteral Obstruction Mice Model

**DOI:** 10.3390/molecules28237690

**Published:** 2023-11-21

**Authors:** Jing Wang, Birui Shi, Yueqing Pan, Zhuan Yang, Wei Zou, Menghua Liu

**Affiliations:** 1 School of Traditional Chinese Medicine, Southern Medical University, Guangzhou 510515, China; wjmjcmgc525@smu.edu.cn; 2 NMPA Key Laboratory for Research and Evaluation of Drug Metabolism & Guangdong Provincial Key Laboratory of New Drug Screening, School of Pharmaceutical Sciences, Southern Medical University, Guangzhou 510515, China; stone0907mua@163.com (B.S.); 13119868617@163.com (Y.P.); yangzhuan1018@163.com (Z.Y.); 3 Changsha Research and Development Center on Obstetric and Gynecologic Traditional Chinese Medicine Preparation, Hunan Provincial Maternal and Child Health Care Hospital, Changsha 410008, China; 4 School of Pharmaceutical Science, University of South China, Hengyang 421001, China

**Keywords:** asperulosidic acid, renal interstitial fibrosis, indoxyl sulfate, OATs, chronic kidney disease

## Abstract

Asperulosidic acid is a bioactive iridoid isolated from *Hedyotis diffusa* Willd. with anti-inflammatory and renal protective effects. However, its mechanism on renal interstitial fibrosis has not been elucidated yet. The present study aims to explore whether asperulosidic acid could retard renal fibrosis by reducing the circulating indoxyl sulfate (IS), which is a uremic toxin and accelerates chronic kidney disease progression by inducing renal fibrosis. In this paper, a unilateral ureteral obstruction (UUO) model of Balb/C mice was established. After the mice were orally administered with asperulosidic acid (14 and 28 mg/kg) for two weeks, blood, liver and kidney were collected for biochemical, histological, qPCR and Western blot analyses. Asperulosidic acid administration markedly reduced the serum IS level and significantly alleviated the histological changes in glomerular sclerosis and renal interstitial fibrosis. It is noteworthy that the mRNA and protein levels of the organic anion transporter 1 (OAT1), OAT3 and hepatocyte nuclear factor 1α (HNF1α) in the kidney were significantly increased, while the mRNA expressions of cytochrome P450 2e1 (Cyp2e1) and sulfotransferase 1a1 (Sult1a1) in the liver were not altered after asperulosidic acid administration. These results reveal that asperulosidic acid could accelerate the renal excretion of IS by up-regulating OATs via HNF1α in UUO mice, thereby alleviating renal fibrosis, but did not significantly affect its production in the liver, which might provide important information for the development of asperulosidic acid.

## 1. Introduction

**Figure 1:**
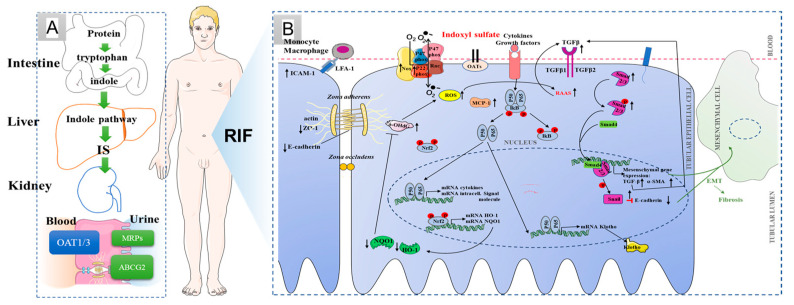
IS production, excretion and its relationship with renal interstitial fibrosis. (**A**) IS processes in the normal body; (**B**) possible mechanism of renal interstitial fibrosis induced by indoxyl sulfate. ↓: Inhibitory effect of IS. ↑: Stimulatory effect of IS.

Chronic kidney disease (CKD) has become a global health problem [1,2]. Renal interstitial fibrosis is the main pathological feature of CKD, subsequently leading to end-stage renal disease if renal interstitial fibrosis is not effectively blocked [3]. With the deepening of our understanding of CKD, urinary toxin has been recognized as an increasingly important factor in the systemic organ dysfunction associated with the progression of CKD [4,5]. Indoxyl sulfate (IS) is one of the most widely studied uremic toxins to date. Clinical research has revealed that IS, as a nephro-vascular toxin, plays an important role in the occurrence and development of renal interstitial fibrosis [5]. On the one hand, IS can promote renal interstitial mononuclear and/or macrophage infiltration; produce a variety of pro-fibrotic factors, such as transforming growth factor β1 (TGF-β1), intercellular adhesion molecule-1 (ICAM-1) and monocyte chemotactic protein-1 (MCP-1); and activate nuclear factor-κB (NF-κB) and/or heat shock protein 90 (HSP90) of renal tubule cells to induce renal interstitial fibrosis [6]. On the other hand, IS induces a large number of oxygen free radicals produced in the process of oxidative stress in renal tubular epithelial cells to promote cell apoptosis, and the apoptosis of renal tubular cells can further develop into renal fibrosis (Figure 1) [7,8]. Furthermore, IS could mediate immune dysfunction to cause vascular endothelial cell damage in patients with end-stage renal disease [9,10]. In CKD patients, plasma IS level, increasing gradually with the progression of CKD stage, has a strong negative correlation with the glomerular filtration rate [11]. For stage III chronic kidney disease, the serum urotoxin is more than 10 times the normal level, while more than 200-fold for uremic patients [11]. Therefore, reducing the accumulation of IS has become an effective treatment for delaying renal fibrosis.

IS in the blood is mainly produced by intestinal bacteria, is metabolized by cytochrome P450 2E1 (CYP2E1) and sulfotransferase 1A1 (SULT1A1) in the liver, and then enters the blood circulation system [12,13]. The excretion of IS mainly depends on renal tubule secretion. The renal organic anion transporter 1 (OAT1) and OAT3, located in the basolateral membrane, is connected with multidrug resistance proteins (MRPs) and ATP-binding cassette transporter subfamily G member 2 (ABCG2) in the apical membrane of proximal tubular cells, which form the excretion channel and mediate the excretion of IS from the blood into the urine [14,15,16]. At present, the strategy of blocking IS mainly includes controlling diet and orally administering intestinal sorbents. However, a low protein intake can easily cause hyperkalemia and malnutrition [17]. Oral sorbents (e.g., AST-120) can adsorb the precursor of indoxyl sulfat from the small intestine to reduce serum concentrations of IS [18]. Harry Sokol et al. pointed out that the metabolism disorder of tryptophan is closely related to inflammatory bowel disease, metabolic syndrome and infectious diseases [19].

*Hedyotis diffusa* Willd. is a traditional Chinese medicine with therapeutic effect for CKD when used as single herb or in combination [20]. Asperulosidic acid, an iridoid compound, has been isolated from *Hedyotis diffusa* Willd [21]. Previously, we confirmed that asperulosidic acid could improve kidney injury, could reduce collagen fiber deposition and exhibits a protective effect on renal interstitial fibrosis rat models induced by unilateral ureteral obstruction (UUO) [22]. However, the effect of asperulosidic acid on urinary toxin accumulation in animals with renal interstitial fibrosis has not been reported. The purpose of this study is to determine whether asperulosidic acid could regulate the synthesis and excretion pathway of IS, thereby delaying the process of renal interstitial fibrosis, and to provide scientific data for the development of asperulosidic acid.

## 2. Results

### 2.1. Quantitative Analysis of IS by the HPLC-MS/MS Method

**Figure 2:**
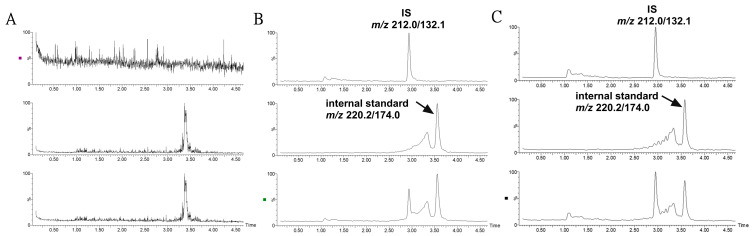
Representative MRM ion chromatograms for the determination of IS and 5-methoxy-2-methyl-3-indoleacetic acid (internal standard) in mouse plasma in a positive ion model. (**A**) Blank plasma; (**B**) blank plasma spiked with IS and internal standard at a low QC sample; (**C**) plasma sample collected at 2 h following the treatment of asperulosidic acid for 2 weeks at a dosage of 14 mg/kg.

The bioanalytical method was validated according to the technical guidelines for the validation of quantitative analysis methods for biological samples from the Pharmacopoeia of People’s Republic of China [23]. The validation parameters were presented in Table S1. As shown in Figure 2, there was no obvious endogenous peak disturbing the detection of IS in mouse plasma. IS had a good linearity in the range of 0.1–100 μg/mL with a correlation coefficient of r = 0.9953. The RSD values for intra- and inter- batch precisions were lower than 14.9% at each quality control (QC) level. The extraction recoveries at three QC concentrations were all above 90.5%, and the matrix effects were in the range of 105.6–115.4%. The stability, including plasma samples at room temperature within 12 h, undergoing three freeze–thaw cycles and at −80 °C for 1 month displayed variability from 85.1% to 108.6%.

### 2.2. Asperulosidic Acid Decreased the Accumulation of IS in UUO Mice

**Figure 3:**
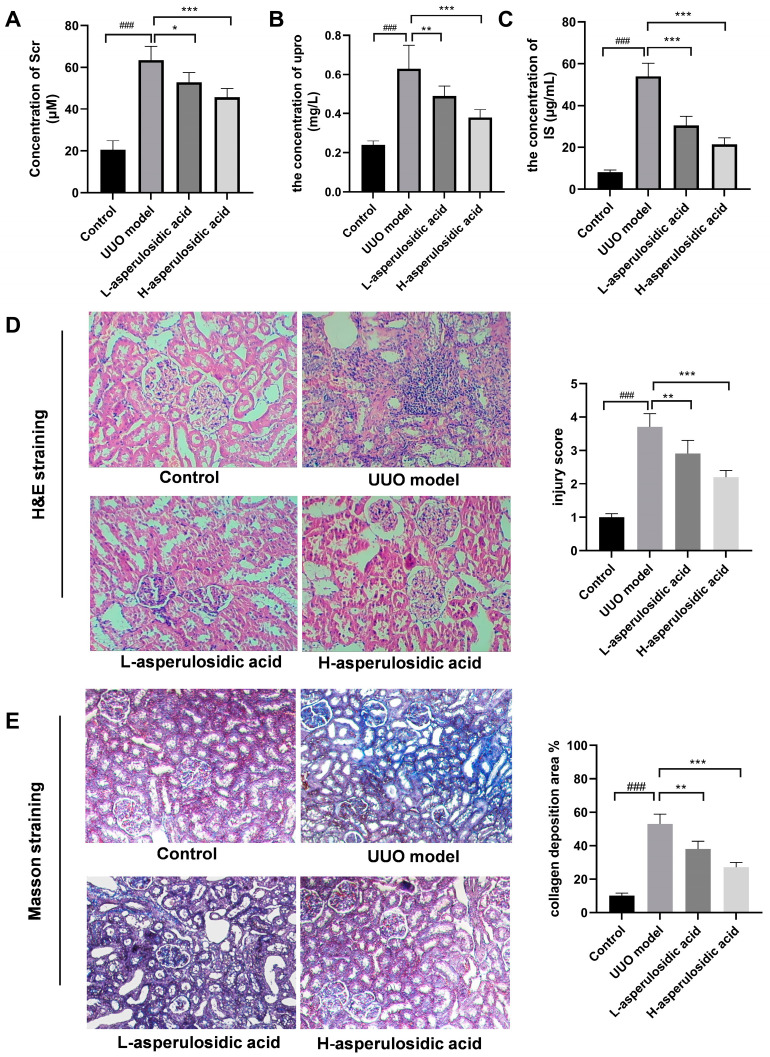
The effects of asperulosidic acid on renal function, histomorphology and IS concentration in UUO mice. (**A**) Serum creatinine (Scr); (**B**) urinary protein (upro); (**C**) H&E-stained kidney sections from each group; (**D**) Masson’s trichrome-stained kidney sections from each group; (**E**) the concentration of IS in plasma collected at 2 h after the final dose of asperulosidic acid. Data are presented as mean ± SD (n = 6). Compared with the control group, ^###^ *p* < 0.001. Compared with the model group, * *p* < 0.05, ** *p* < 0.01 and *** *p* < 0.001.

A mouse model of renal interstitial fibrosis was successfully established by UUO. With the extension of the obstruction time, the levels of serum creatinine (Scr) and urinary protein (upro) were significantly increased (Figure 3A,B). After treatment with asperulosidic acid (14 and 28 mg/kg), Scr and upro were reduced by 17–28% and 33–52%, respectively. Both H&E and Masson staining results revealed that a large number of inflammatory cells were infiltrated in the mouse kidney tissue and collagen fibers were deposited in the renal interstitium in the UUO model, but this pathological injury could be improved after the oral administration of asperulosidic acid at two dosages. The IS content in the mice plasma was detected by HPLC-MS/MS. It is worth noting that the IS content in UUO mice model was significantly increased. Compared with the model group, asperulosidic acid treatment at 14 and 28 mg/kg reduced IS content by 19–42% (Figure 3C). All these results suggest that asperulosidic acid can improve the renal fibrosis of UUO mice by inhibiting the accumulation of IS in UUO mice.

### 2.3. Effects of Asperulosidic Acid on the Liver Metabolic Enzymes of IS in UUO Mice

**Figure 4:**
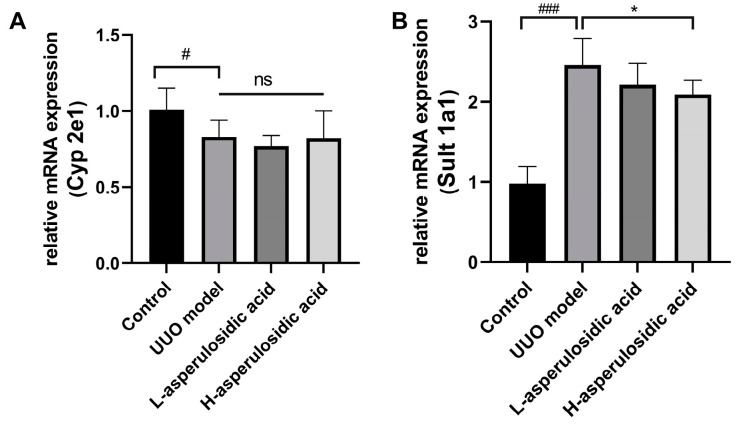
The effects of asperulosidic acid on the mRNA expression of Cyp2e1 and Sult1a1 after oral treatment for 2 weeks. (**A**) mRNA expression of Cyp2e1; (**B**) mRNA expression of Sult1a1. Data are presented as mean ± SD (n = 6). Compared with the control group, ^#^ *p* < 0.05, ^###^ *p* < 0.001. Compared with the model group, * *p* < 0.05. ns: no significant difference.

The mRNA expression of Cyp2e1 and Sult1a1 were examined to evaluate whether asperulosidic acid could act on the increase in IS synthesis in liver. As shown in Figure 4, the mRNA expression of Cyp2e1 was decreased by a certain extent, whereas a significant increase in Sult1a1 was observed in UUO mice on the 14th day post-surgery. However, after the administration of asperulosidic acid, the Cyp2e1 mRNA expression had no significant change in the two treatment groups. Interestingly, the mRNA expression of Sult1a1 was reduced by 18.4% after treatment with asperulosidic acid at the high dose of 28 mg/kg, as shown in Figure 4B. These data indicate that a high concentration of asperulosidic acid could reduce the synthesis of IS in the liver to some extent by down-regulating Sult1a1.

### 2.4. Effects of Asperulosidic Acid on the Excretion Pathway of IS in UUO Mice

**Figure 5:**
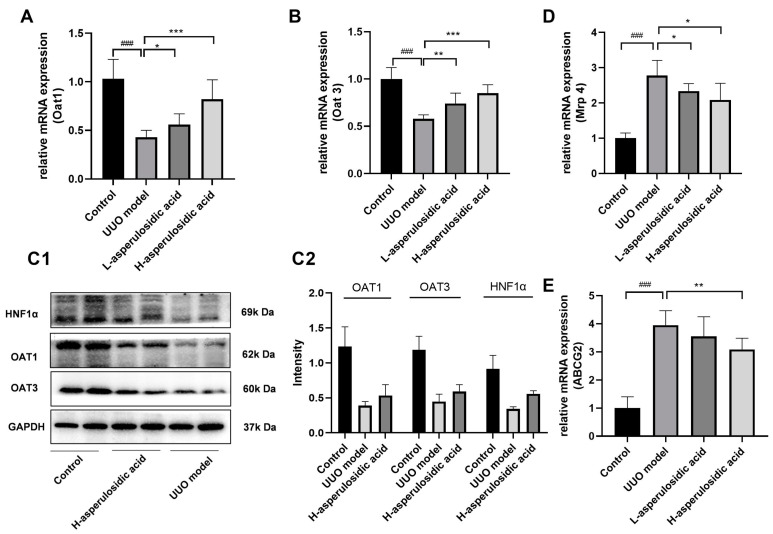
The effects of asperulosidic acid on the excretion pathway of IS in UUO mice. (**A**,**B**) mRNA expression of Oat1 and Oat3 in kidneys (n = 6). (**C1**) Protein bands of HNF1α, OAT1 and OAT3 in the fibrotic renal tissue (n = 2). (**C2**) Semi-quantitative analysis of HNF1α, OAT1 and OAT3 (mean ± average deviation). (**D**,**E**) mRNA expression of Mrp4 and Abcg2 in kidneys (n = 6). Data are presented as mean ± SD. Compared with the control group, ^###^ *p* < 0.001. Compared with the model group, * *p* < 0.05, ** *p* < 0.01 and *** *p* < 0.001.

The mRNA or/and protein levels of OAT1, OAT3, MRP4 and ABCG2 were determined to evaluate whether asperulosidic acid acted on the excretion pathway of IS, thereby reducing the plasma concentration of IS. The qPCR and Western blot results show that the expressions of kidney OAT1 and OAT3 were significantly inhibited during the fibrotic progression (Figure 5A–C), whereas the mRNA expressions of Mrp4 and Abcg2 were significantly elevated (Figure 5D,E). The reduced expressions of OATs indicated an accumulation of urinary toxin IS in the blood, as they were the key uptake proteins of IS that mediate the excretion of IS from the blood into the urine. After the oral administration of asperulosidic acid at two dosages, the expressions of OATs were significantly increased compared with those in the model group. These data indicate that the improvement effect of asperulosidic acid in renal interstitial fibrosis is closely related to the up-regulations of OATs to promote IS clearance.

### 2.5. Effect of Asperulosidic Acid on the Hepatocyte Nuclear Factor 1α (HNF1α)

**Figure 6:**
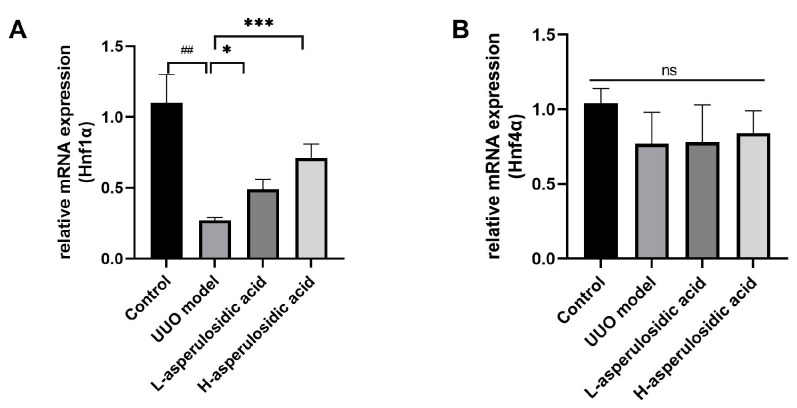
The effects of asperulosidic acid on the expressions of Hnf4α and Hnf1α. (**A**) mRNA expression of Hnf1α in kidneys (n = 6). (**B**) mRNA expression of Hnf4α in kidneys (n = 6). Compared with the control group, ^##^ *p* < 0.05. Compared with the model group, * *p* < 0.05 and *** *p* < 0.001. ns: no significant difference.

It is known that transcription factors, such as HNF1α and HNF4α, play key roles in the regulation of the expression of OATs. As shown in Figure 6, the mRNA expression of Hnf1α, rather than that of Hnf4α, was reduced by 76.8% in UUO mice (Figure 6A). After asperulosidic acid treatment at 14 mg/kg and 28 mg/kg, the expression was increased by 1.8-fold and 2.6-fold compared with that in the model group, respectively. The protein levels in the Western blot analysis were consistent with mRNA levels (Figure 5C and Figure 6B). The expression of Hnf4α decreased slightly in the model group, but there was no significant difference. In addition, asperulosidic acid at two doses did not alter the expression of Hnf4α (Figure 6B). This demonstrates that asperulosidic acid can up-regulate OAT protein expressions via increasing HNF1α to accelerate the excretion of IS and then alleviates the progression of renal fibrosis.

## 3. Discussion

CKD has become a major disease worthy of global attention, both because of its high morbidity and mortality and it is a major predisposing factor for cardiovascular disease. According to a global survey in 2017, there were 697.5 million CKD patients worldwide, about one-third of whom were in China and India [24]. With the failure of renal function in CKD patients, a variety of metabolites, namely uremic toxins, are difficult to excrete and thus accumulate in the body, causing toxicity, which results in uremia [25]. To date, urinary toxin retention has been recognized as an increasingly important factor in systemic organ dysfunction that accompanies the progression of CKD [26]. Dialysis is an effective method to remove uremic toxins [27]. But in fact, IS, as a protein-bound small-molecule uremic toxin, cannot be cleared by ordinary dialysis and high-throughput dialysis membranes [28]. Therefore, reducing IS has become a potential therapeutic target for controlling the development of CKD.

In normal kidneys, IS enters the renal tubular epithelial cells from the blood, mainly through OATs, and is actively secreted into the urine by MRPs and ABCG2 [28]. On the contrary, the inhibition of OATs can cause different degrees of kidney injury, such as those caused by cisplatin, arsenic and mercury [29,30]. In an Oat1^−/−^ model, multiple uremic toxins cannot be excreted in time, resulting in increased plasma concentrations and accelerated CKD development [31]. In this paper, we found that, with the increase in the plasma concentration of IS, there was a decreasing trend of OAT1/3 expression during the renal fibrosis process, while the expressions of MRP4 and ABCG2 increased, indicating that the accumulation of IS is directly related to the decrease in OAT expression. This result is consistent with those of previous reports [28,32].

OAT expression is regulated by a variety of transcription factors, such as liver X receptor (LXR), HNF1α and HNF4α [33,34,35]. It has been reported that LXR activation downregulates OAT1 expression in the renal proximal tubule [33]. However, mRNA expressions were considerably LXR-downregulated with prolonged obstruction (Figure S1). In the kidney, HNF1α is expressed in proximal tubule epithelial cells. The tissue-specific expression of OAT1 is regulated by HNF1α, mainly because the OAT1-promoter region contains HNF1-binding sites [34]. Meanwhile, HNF4α could markedly transactivate the OAT1 promoter via an inverted repeat separated by eight nucleotides (IR-8) [36]. In this paper, we confirmed that the expression of HNF1α, rather than that of HNF4α, was decreased after 14 days of obstruction in UUO mice by qRT-PCR and Western blot methods, indicating that a decreased HNF1α expression mediates a decreased tissue-specific OAT1/3 expression.

Asperulosidic acid, a bioactive compound obtained from *Hedyotis diffusa* Willd., has a good efficacy in the treatment of renal diseases, especially in anti-renal fiber activity. Previously, we proved that asperulosidic acid could significantly reduce plasma Scr, BUN, UA and upro, and inhibit the expression of fibrotic factors, including TGF-β1, α-SMA, collagenIII and fibronectine. Its mechanism of action is closely related to inhibiting the NF-κB and TGF-β1/smad2/smad3 signaling pathways [22]. In this study, it was found that the content of IS decreased significantly after the administration of asperulosidic acid. Aiming at the production and excretion of indoxyl sulfate, we analyzed the causes of the accumulation of indoxyl sulfate in UUO mice and clarified the mechanism of reducing IS by asperulosidic acid. The results suggest that asperulosidic acid does not significantly affect the synthesis of IS in the liver, but significantly up-regulates the expression of OAT1 through HNF1α and then accelerate the excretion of IS, thereby delaying renal interstitial fibrosis. To clarify the extent of asperulosidic acid’s contribution to the observed effects, a rescue experiment using probenecid (an OAT inhibitor) in conjunction with asperulosidic acid was conducted (Figure S2). After asperulosidic acid oral treatment for 2 weeks, probenecid was administered intravenously at a dose of 200 mg/kg [37]. Compared with the H-asperulosidic acid group, probenecid increased the plasma IS concentration by 2.4-fold. This is new evidence of the anti-renal fiber effect of asperulosidic acid. In addition, we further investigated how asperulosidic acid activates the protein level of HNF1α. A previous study has shown that the mitogen-activated protein kinase (MAPK) signal leads to increased extracellular signal-regulated kinase (ERK) 1/2 phosphorylation, and ERK1/2 activation inhibits the protein levels of HNF1α, indicating that the MPAK signal is a negative regulator of HNF1α in all three EBV-infected liver cell models [38]. The MAPK signaling pathway is one of the recognized pathological mechanisms of renal interstitial fibrosis. Its subgroups, such as p38 MAPK, ERK and c-Jun N-terminal kinase (JNK), mediate signal transduction in fibrosis [39]. Previously, we reported that the asperulosidic acid process has an obvious inhibitory effect on the MAPK signaling pathway, especially causing a decrease in ERK1/2 phosphorylation in LPS-induced RAW264.7 macrophages [21]. Based on the above facts, we hypothesized that the activation of HNF1α by asperulosidic acid is probably related to its inhibition effect on the MAPK pathway. Unfortunately, to date, the relationship among HNF1α, NF-κB and TGF-β1 was unclear, and a further experiment to confirm it using multi-omics technology will be conducted in order to understand comprehensively the benefits of asperulosidic acid in renal fibrosis improvement.

## 4. Materials and Methods

### 4.1. Chemicals and Regents

Authentic standards of IS potassium salt and 5-methoxy-2-methyl-3-indoleacetic acid (internal standard) were purchased from Sigma Aldrich (Shanghai, China) Trading Co., Ltd. (Shanghai, China). Asperulosidic acid (98.5% purity; HPLC analysis) was purchased from the Fuzhou Biological Technology Company (Linchuan, China). The total RNA isolation kit was obtained from Foregene Co., Ltd. (Chengdu, China). The kits of serum creatinine (Scr) and urinary protein (upro) were provided by the Nanjing Jiancheng Bioengineering Institute (Nanjing, China). Acetonitrile and formic acid were of gradient-grade from Merck & Co., Inc. (Merck KGaA, Darmstadt, Germany). All other chemical reagents are commercially available.

### 4.2. Animal Experiment

Male Balb/C mice, of 18–20 g body weight, were provided by the Experimental Animal Center of Southern Medical University. All mice were fasted for 12 h before surgery. After being anesthetized with chloral hydrate at a dose of 0.3 mg/kg, ureteral ligation was performed as described previously [40]. After the mice woke up, the activity state of the mice was observed, and penicillin was injected on the day after the modeling. Twenty-four mice were randomly divided into four groups: a sham group, a UUO model group, and two asperulosidic acid treatment groups (14 and 28 mg/kg) formed according to previous research [22].

After 14 days of administration, the mice were placed in a metabolic cage where urine was collected for 24 h and placed at −80 °C for upro quantification. Blood was collected from the orbital veins of mice, and the whole blood was centrifuged at 5000 rpm for 5 min. Plasma was collected at 2 h and stored in the refrigerator at −20 °C for the detection of creatinine and IS. After dissecting all mice, the left and right kidneys were removed and separated. Part of the right kidney was fixed in 4% paraformaldehyde for histology analysis using the hematoxylin and eosin (H&E) and Masson’s Trichrome staining procedures. The remaining material was frozen in liquid nitrogen for the detection of mRNA and protein levels.

### 4.3. Histology Analysis

The kidneys of each group were embedded in paraffin wax and then sliced into 4 μm slices. The procedure of H&E staining and Masson’s Trichrome staining was performed as previously reported [22]. The extent of renal injury was determined by the size of the glomeruli and the changes in the renal tubules with H&E staining. Histopathological changes were graded from 0 to 4: 0, normal; 1, <25% changes; 2, 25–50% changes; 3, 50–75% changes; and 4, >75% changes. In the result of Masson’s staining, the extent of renal interstitial fibrosis was assessed based on the amount of collagen deposition (blue area was a percentage of the total cortical area). All pathological images were quantified by the Image-Pro Plus 6.0 software (Media Cybernetics, Silver Spring, MD, USA).

### 4.4. RT-qPCR

Total RNA was extracted from the kidney tissue by the Trizol method following the kit instructions [41]. The kidney tissue was ground into a powder in liquid nitrogen, 1 mL of pre-cooled Trizol reagent was added, and then the tissue transferred to the centrifugal tube. The samples were centrifuged at 4 °C, 12,000 rpm for 15 min. Then, the upper layer was taken, same amount of isopropyl alcohol was added, and the layer was stored at −20 °C for 30 min. After the supernatant was discarded, the sample was rinsed with 1 mL of 75% ethanol. The residue was dissolved with 30 μL RNase-free ddH_2_O for RNA concentration determination. The genes were detected by RNA reverse transcription and quantitative real-time PCR (RT-qPCR). The components of the reverse transcription reaction system included 4 μL of 5 × PrimeScript RT Master Mix and 1000 ng of total RNA. The reaction volume was 20 μL. The cDNA reaction conditions were as follows: 37 °C for 15 min and 85 °C for 5 s. The primer sequences for Cyp2e1, Sult1a1, Oat1, Oat3, Mrp4, Abcg2, Hnf1α, Hnf4α and Gapdh were designed as shown in Table 1 [42,43,44,45]. The PCR reaction conditions were: 95 °C for 30 s, 95 °C for 3 s and 60 °C for 30 s. A total of 40 cycles were performed. The threshold cycle (C_t_) was recorded by the instrument’s software (7500 Fast System Software version v2.3). The fold changes of each target gene at the mRNA level were calculated according to the g 2^−ΔΔCt^ method.

### 4.5. Western Blotting

The total protein of renal tissue was extracted by Cell Signaling Technology [41]. The protein sample was added to the pre-cooled protein cracking buffer at the ratio of 1 mL/0.1 g, homogenized for 15 min, and centrifuged at 10,000 g/min for 15 min. The supernatant was determined using the Bradford method. The protein samples were loaded onto 10% SDS-PAGE for 1 h and then transferred to the PVDF membrane. The membrane was blocked with 5% non-fat milk in TBS-T for 2 h at room temperature and then incubated overnight with the primary antibodies, including OAT1 (1:1000), OAT3 (1:1000), HNF1α (1:1000; ABdonal) and GAPDH (1:1000; Cell Signaling Technology, Danvers, MA, USA), at 4 °C. In view of the similar molecular weight of OAT1 and OAT3, membrane stripping was performed according to a previous report [46]. For OAT1, the membranes were incubated firstly with OAT1 primary antibody overnight and then incubated with the corresponding secondary antibody at room temperature for 2 h. The blots were scanned using a Fluorchem R multifunctional imaging analysis system. The membranes were then stripped using stripping buffers at room temperature for 2 h to remove the bound primary and secondary antibodies. After stripping, the membranes were washed with 1 × TBST buffer solution three times. A new round of incubation was conducted using primary and secondary antibodies of OAT3. All blots were detected using the enhanced chemiluminescence (ECL) method. The target proteins were analyzed using a gel image processing system (FluorChem R, ProteinSimple, Silicon Valley, CA, USA).

### 4.6. Detection Method of IS

The plasma sample preparation method was modified according to a previous report [47]. An aliquot of 50 μL mouse plasma was transferred to a 1.5 mL tube, then 10 μL internal standard solution and 600 μL acetonitrile were mixed. After being centrifuged at 10,000 rpm for 15 min, the supernatant of 250 μL was transferred into a new 1.5 mL tube. After adding 250 μL distilled water, the solution was centrifuged at 13,000 rpm for 20 min. Finally, 5 μL of the supernatant was injected into the LC–MS/MS for analysis.

The concentration of IS in the mouse plasma was determined by the LC-MS/MS method, which consists of a Waters Acquity UPLC system and a Quattro Premier XE triple quadrupole mass spectrometer (Waters Corporation, Milford, MA, USA). The chromatographic separation was performed on a Waters UPLC T3 (100 × 2.1 mm, 1.8 μm) column. The mobile phase was composed of 0.1% formic acid (A) and acetonitrile (B) at the flow rate of 0.3 mL/min by a gradient elution procedure as follows: 0–1.5 min, 5% B; 1.5–2 min, 5–95% B; 2–3 min, 95% B; 3–3.1 min, 5–95% B; 3,1–5 min, 5% B. The column temperature was 25 °C. Detection was performed in the positive ion mode using multiple reaction monitoring (MRM) of the *m/z* 212.0/132.1 for IS and *m/z* 220.2/174.0 for the internal standard. The working parameters of the mass spectrum were as follows: capillary voltage: 1.0 kV; ion source temperature: 105 °C; desolvent gas (N_2_) temperature: 350 °C; desolvent gas (N_2_) flow rate: 600 L/h; cone-hole airflow (N_2_) flow rate: 50 L/h; and impact gas flow rate: 0.12 mL/min.

The calibration range was converted to a plasma concentration of 0.1–100.0 μg/mL for IS. Three QC standards were prepared at 0.25, 50.0 and 80.0 μg/mL. The lower limit of quantification was a signal-to-noise ratio of 10. The inter-batch and intra-batch precision, accuracy, matrix effect, recovery and stability were verified with OC samples at three concentrations and at least 6 samples for each concentration. Analytical data were obtained from the Masslynx V4.1 software (Waters Corporation, Milford, MA, USA).

### 4.7. Statistical Analyses

The data obtained in the experiment were analyzed by the GraphPad prism 8 software (Boston, MA, USA) and expressed as the mean value ± standard deviation (SD). The difference between each group was analyzed by a one-way ANOVA of SPSS 18.0 software (Chicago, IL, USA). *p* < 0.05 was regarded as statistically significant.

## 5. Conclusions

The effective blocking of renal interstitial fibrosis is the key step in treating CKD and delaying the progression of CKD to end-stage renal disease. Asperulosidic acid has been shown to be effective in ameliorating renal interstitial fibers in UUO mice. Together with the effects of asperulosidic acid on the NF-κB and TGF-β1 signaling pathways reported earlier, it is a new finding that asperulosidic acid can up-regulate OAT expression via HNF1α and promote the excretion of uremic toxin IS, thus delaying the process of renal interstitial fibrosis. This provides new evidence for the development and clinical application of asperulosidic acid.

## Figures and Tables

**Table 1:** The primer sequences of the target genes.

Name	Forward Primer	Reverse Primer	Reference
*Cyp2e1*	CTTTGCAGGAACAGAGACCA	ATGCACTACAGCGTCCATGT	[42]
*Sult1a1*	CACAAGGGTCCTCTCCTTAGC	TGACAGCGGAACGTGAAGTC	[43]
*Oat1*	CTACTGCATTTTCCGGCTCC	ATAGGCACGGGTGTGAATAGG	[44]
*Oat3*	ACAGCAGCCCTTCATCCCTAATG	CCTCCCAGTAGAGTCATGGTCAC	[44]
*Mrp4*	AGAGGCCATCGTCAGCATTC	ACTGTCTAGTGCCTTGTCCC	[44]
*Abcg2*	TGGCTGTCCTGGCTTCAGTAC	CCAAGAATTCATTATACTGCAA	[44]
*Hnf1α*	GCCACCATGGTTTCTAAGCTGAGC	GGATCCCTGGGAAGAGGAGGC	[45]
*Hnf4α*	GGATATGGCCGACTACAGCG	AGATGGGGACGTGTCATTGC	designed
*Gapdh*	CTTTGGCATTGTGGAAGGGC	TGCAGGGATGATGTTCTGGG	[44]

## Data Availability

Data are contained within the article.

## Supplementary Materials

The following supporting information can be downloaded at: https://www.mdpi.com/article/10.3390/molecules28237690/s1. Figure S1: mRNA expression of Lxr in UUO mice; Figure S2: The concentration of IS in the mouse plasma after the final administration. Table S1: Methodology of IS.
