# Supplementary material for: Asperulosidic Acid Ameliorates Renal Interstitial Fibrosis via Removing Indoxyl Sulfate by Up-Regulating Organic Anion Transporters in a Unilateral Ureteral Obstruction Mice Model
Source: Molecules. 2023 Nov 21;28(23):7690. doi: 10.3390/molecules28237690 (PMC10707915; doi:10.3390/molecules28237690)
Supplement: Supplementary file 1 [file molecules-28-07690-s001.zip › molecules-2666314-supplementary.pdf]

Table S1. The validation parameters for IS quantitation in mouse plasma at three concentrations (n=6)

Concertation (μg/mL)	Precisions (%)		Accuracy (%)	Matrix effect (%)	Recovery (%)	Stability (%)		
	intra-batch	inter-batch				at room temperature within 12 h	undergoing 3 freeze–thaw cycles	at -80 ºC for 1 month
0.25	14.9%	12.1%	105.4%	115.4%	90.5%	90.4%	89.4%	85.1%
50.0	10.4%	6.4%	98.5%	107.2%	94.2%	96.6%	95.6%	108.6%
80.0	7.8%	5.3%	97.4%	105.6%	93.7%	95.1%	101.8%	90.4%

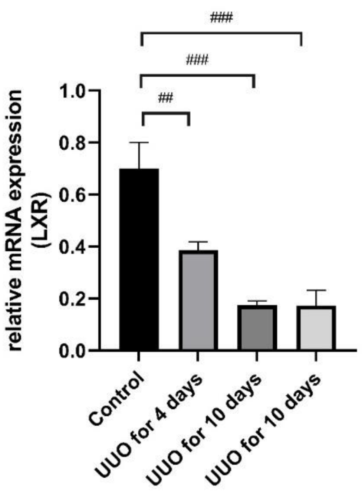

Figure S1. the mRNA expression of Lxr in UUO mice (n=6). Samples were detected on 4th, 10th and 14th days. Compared with control group: $^{##}$ $p < 0.01$, $^{###}$ $p < 0.001$.

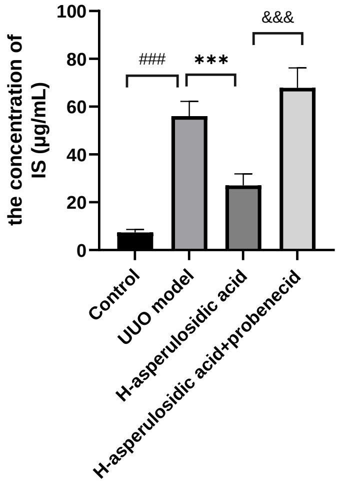

Figure S2. The concentration of IS in mouse plasma after the last administration at 2 h (n=4). H-asperulosidic acid: UUO mice were oral asperulosidic acid for 14 days at 28 mg/kg. H-asperulosidic acid+probenecid: after the last administration of asperulosidic acid on the 14th day, probenecid was administered intravenously with a dose of 200 mg/kg. Compared with control group: $^{###}$ $p < 0.001$. Compared with UUO group: *** $p < 0.001$. Compared with H-asperulosidic acid group: $^{\&\&\&}$ $p < 0.001$.
